# Usefulness of axonal tract-dependent OCT macular sectors for evaluating structural change in normal-tension glaucoma

**DOI:** 10.1371/journal.pone.0185649

**Published:** 2017-10-03

**Authors:** Kazuko Omodaka, Tsutomu Kikawa, Yukihiro Shiga, Satoru Tsuda, Yu Yokoyama, Haruka Sato, Junko Ohuchi, Akiko Matsumoto, Hidetoshi Takahashi, Masahiro Akiba, Toru Nakazawa

**Affiliations:** 1 Department of Ophthalmology, Tohoku University Graduate School of Medicine, Sendai, Japan; 2 Department of Ophthalmic Imaging and Information Analytics, Tohoku University Graduate School of Medicine, Sendai, Japan; 3 Topcon Corporation, Tokyo, Japan; 4 Department of Medicine, Division of Ophthalmology, Tohoku Medical and Pharmaceutical University, Sendai, Japan; 5 Department of Retinal Disease Control, Ophthalmology, Tohoku University Graduate School of Medicine, Sendai, Japan; 6 Department of Advanced Ophthalmic Medicine, Tohoku University Graduate School of Medicine, Sendai, Japan; Massachusetts Eye & Ear Infirmary, Harvard Medical School, UNITED STATES

## Abstract

**Purpose:**

To identify sectors of the optical coherence tomography (OCT) macular map that could be used to effectively assess structural progression in patients with normal-tension glaucoma (NTG).

**Methods:**

This study examined 117 eyes of 117 NTG patients to establish axonal tract-dependent macular sectors, and also examined a separate group of 122 eyes of 81 NTG patients to evaluate the ability of these sectors to reveal glaucoma progression. Longitudinal data, including macular maps from at least 5 OCT examinations performed over at least 2 years, was available for all patients in this group. Circumpapillary retinal nerve fiber layer thickness (cpRNFLT), temporal clockwise sector scans (from 7 to 11 o’clock), macular retinal nerve fiber layer thickness (mRNFLT), and macular ganglion cell layer plus inner plexiform layer thickness (mGCIPLT) were measured with spectral-domain OCT (3D OCT-2000, TOPCON). The axonal tract-dependent macular sectors were identified by calculating Spearman’s rank correlation coefficient for each point on a grid overlaid on the macular map and cpRNFLT in each clockwise scan sector. Trend and event analyses for the slope of progression in each sector and macular map were performed. Visual field progression in the macula was defined by the presence of more than 2 progressive test points in the 16 central test points of the Humphrey field analyzer SITA standard 24–2 program, evaluated with Progressor software.

**Results:**

The slope of progression in the entire macular area was -0.22 ± 0.58 μm/year for mRNFLT and -0.35 ± 0.52 μm/year for mGCIPLT. The fastest-progressing mRNFLT sector (-1.00 ± 0.84 μm/year, p < 0.001) and mGCIPLT sector (-1.16 ± 0.63 μm/year, p < 0.001) progressed significantly faster than the overall macula. Classifying patients according to visual field progression showed that baseline mRNFLT in the inferior hemifield, 7 and 8 o’clock sectors, as well as baseline mGCIPLT in the overall macular map, inferior hemifield, and 8 o’clock sector, were significantly lower in progressors (22 eyes) than non-progressors (100 eyes). There were significant differences in mRNFLT slope in 8 o’clock sector and in the fastest progressing sector in progressors and non-progressors, but mGCIPLT did not differ, even in the fastest-progressing sector. Event analysis showed that progression occurred most frequently in inferior mRNFLT and superior mGCIPLT in this study.

**Conclusion:**

Axonal tract-dependent OCT macular sectors could effectively reveal structural change in patients with NTG. Furthermore, mRNFLT slope was consistent with visual field progression. This method promises to open new avenues for the OCT-based evaluation of glaucoma progression.

## Introduction

Glaucoma is the second most common cause of blindness worldwide [1,2] and is an increasingly serious issue in aging societies [2], as aging is an important risk factor for glaucoma progression. [3] Glaucoma is a type of optic neuropathy characterized by optic disc cupping and optic nerve fiber degeneration with corresponding visual disturbance. [4] Lowering intraocular pressure (IOP) is an effective, evidence-based treatment for open-angle glaucoma (OAG), [5,6]. However, when IOP is in the normal range, as in normal-tension glaucoma (NTG), the most common type of OAG in some locations, most notably Asia [7–9], IOP-lowering therapy is less effective. IOP control is still important for the management of NTG, but IOP is not a good biomarker of disease progression in NTG. Thus, daily clinical practice requires alternative methods of analyzing glaucoma progression in NTG in order to preserve the quality of life (QOL) of patients.

Macular visual function is particularly important for QOL, and macular lesions are more common in NTG patients. [10,11] The optic nerve head is most susceptible to glaucomatous lesions in the lower nasal area and in the axonal tract, connected to the central 10° (radius) of the macular area. [12–14] The gold standard for analyzing the progression of glaucoma is visual field analysis with the Humphrey field analyzer (HFA), using the 24–2 or 30–2 programs. However, due to the 6° distance between each test point in these HFA programs, the central radial area, within 4.2° of the fixation, contains only 4 test points. Thus, HFA is relatively less sensitive to visual field loss caused by macular lesions in glaucoma. On the other hand, the HFA 10–2 test pattern has 68 test points in the macular area and thus may be a better choice to evaluate glaucoma-associated macular damage. However, it is hard to perform both the 24–2 and 10–2 tests on the same day, due to patient fatigue and time management requirements in clinical practice. Previously, we proposed methods of saving time during visual field testing by using fewer test points in the macular area [15] or by using optical coherence tomography (OCT) macular maps to simulate the results of the 10–2 visual field test program [16]. However, methods to reveal longitudinal changes in macular structure remain lacking. Therefore, the management of glaucoma, especially of NTG, requires better examination methods for the macula.

Generally, structural changes precede functional changes in glaucoma. [17–19]. OCT has proven to be useful for the diagnosis of glaucoma and for following disease progression, particularly OCT measurements of circumpapillary retinal nerve fiber layer thickness (cpRNFLT). [20] Low cpRNFLT [21] or the rapid loss of cpRNFLT [22] indicate visual field loss in patients with preperimetric glaucoma (PPG). Furthermore, spectral domain (SD)-OCT has allowed the use of segmentation algorithms that can separate each retinal layer in the macular area and allow us to measure the macular retinal nerve fiber layer (mRNFL) and ganglion cell layer plus inner plexiform layer (mGCIPL) separately. [23,24] These macular layer measurements have the same performance in glaucoma diagnosis as cpRNFLT. [25,26] Interestingly, patients with decreased baseline thickness in the macular ganglion cell complex have a steeper mean deviation (MD) slope, indicating faster progression. [27] Nevertheless, although OCT measurements of the macula are commonly used to detect glaucoma, they are not usually used to evaluate glaucoma progression, despite their potential usefulness for this purpose.

This study measured the correlations between mRNFL thickness (mRNFLT), mGCIPL thickness (mGCIPLT) and cpRNFLT at each point on a grid overlaid on OCT scans of the macula, identified clusters of abnormal points, and then evaluated the potential of these clusters to reveal structural changes in the macula. Previously, we reported that sectors of the macular map could reveal structural changes in eyes with mild or moderate NTG (101 eyes) [28]. Here, we extended this analysis with a new data set of 117 eyes of 117 cases that included cases of advanced NTG, and identified sectors of the macular map that depended on the axonal tract. Then, we evaluated thinning of the mRNFL and mGCIPL in the overall macular map, in each hemifield, and in the temporal clockwise sectors of 122 eyes with glaucoma. Macular maps from at least 5 OCT examinations performed over a period of at least 2 years were available for all subjects. Finally, we analyzed these data with trend and event analyses. Based on our results, we propose a new, objective, OCT-based method to evaluate glaucoma progression, and we hope that this method will open new avenues for more sensitive assessments of glaucoma progression.

## Material and methods

### Inclusion criteria

Identification of axonal tract-dependent macular sectors was performed in a group of 117 eyes of 117 Japanese patients with mild (n = 55), moderate (n = 32), and severe NTG (n = 30). The inclusion criteria were a diagnosis of NTG (untreated IOP < 22 mmHg), spherical equivalent (SE) refractive error > -8.00 diopters (i.e., excluding patients with high myopia), and a glaucomatous visual field matching the Anderson-Patella classification. The exclusion criteria were (1) decimal visual acuity < 0.3, (2) the presence of other retinal macular diseases, such as macular hole, premacular fibrosis, and age-related macular degeneration, (3) concomitant ocular disease and systemic disease affecting the visual field, and (4) cataract progression. Investigation of the potential of the axonal tract-dependent macular sectors to reveal glaucoma progression was performed in newly recruited group of 122 eyes of 81 NTG patients, for all of whom at least 5 reliably measured OCT macular maps (with image quality >40) were available. The inclusion and exclusion criteria were the same as described above.

This study adhered to the tenets of the Declaration of Helsinki, and the protocols were approved by the Clinical Research Ethics Committee of the Tohoku University Graduate School of Medicine (study 2014-1-805). Participants provided their written informed consent to participate in this study. The ethics committee also approved this consent procedure.

### OCT examination

Maps of mRNFLT, mGCIPLT and cpRNFLT were derived from macular cube scans of a 6 x 6 mm square area that corresponded to the central 20 degrees. The maps were centered on the fovea and captured with 3D OCT-2000 software (ver. 8.00, Topcon Corporation, Tokyo, Japan). A 10 x 10 grid comprising 100 points was laid over the maps for the analysis. Scans were excluded if the image quality was less than 70 or if segmentation in the images was inaccurate. Every scan was manually checked for segmentation errors and was excluded if errors were found. Images that were not accurately centered on the macula were manually adjusted.

### Visual field examination

Progression of the HFA 24-2-measured visual field (VF) was analyzed with Progressor software (Nidek, Japan). Values from two successive VF test results from within one year before entry into this study were used as a baseline. Test points with a slope of visual sensitivity loss of worse than -1 dB/year were defined as progressive. Patients with more than 2 continuous progressive test points within the 16 central points during the 2 years of the study were included in the VF progression group. [29,30]

### OCT-measured progression

After identification of the axonal tract-dependent macular sectors, the slope of progression for average retinal layer thickness loss (for mRNFLT and mGCIPLT) was calculated. In the trend analysis, if the slope of change in a particular scan sector was statistically significantly negative (p < 0.05) in comparison to the natural course in a normative database, [31] it was regarded as progressive. Furthermore, for the event analysis, baseline thickness was set according to the two initial examinations. If a measured value was significantly lower than the natural fluctuation level of two averaged baseline values, the sector was regarded as progressive. When we detected progression in the subsequent continuous data, we defined the patient as progressive on the first examination day.

### Statistical analysis

Spearman’s rank correlation coefficient was used to determine whether mRNFLT and mGCIPLT in each grid point were correlated with cpRNFLT in each temporal clockwise sector (i.e., the 7, 8, 9, 10, and 11 o’clock sectors). An OCT grid point was defined as correlated with a particular clockwise cpRNFLT sector when the highest correlation coefficient was with that sector. The statistical analysis used the Wilcoxon signed-rank test for the superior and inferior hemifields, and Fisher’s exact test for the grid points. Differences between groups were assessed with the Kruskal-Wallis test. The McNemar test was used for frequency data on sex.

## Results

**Fig 1:**
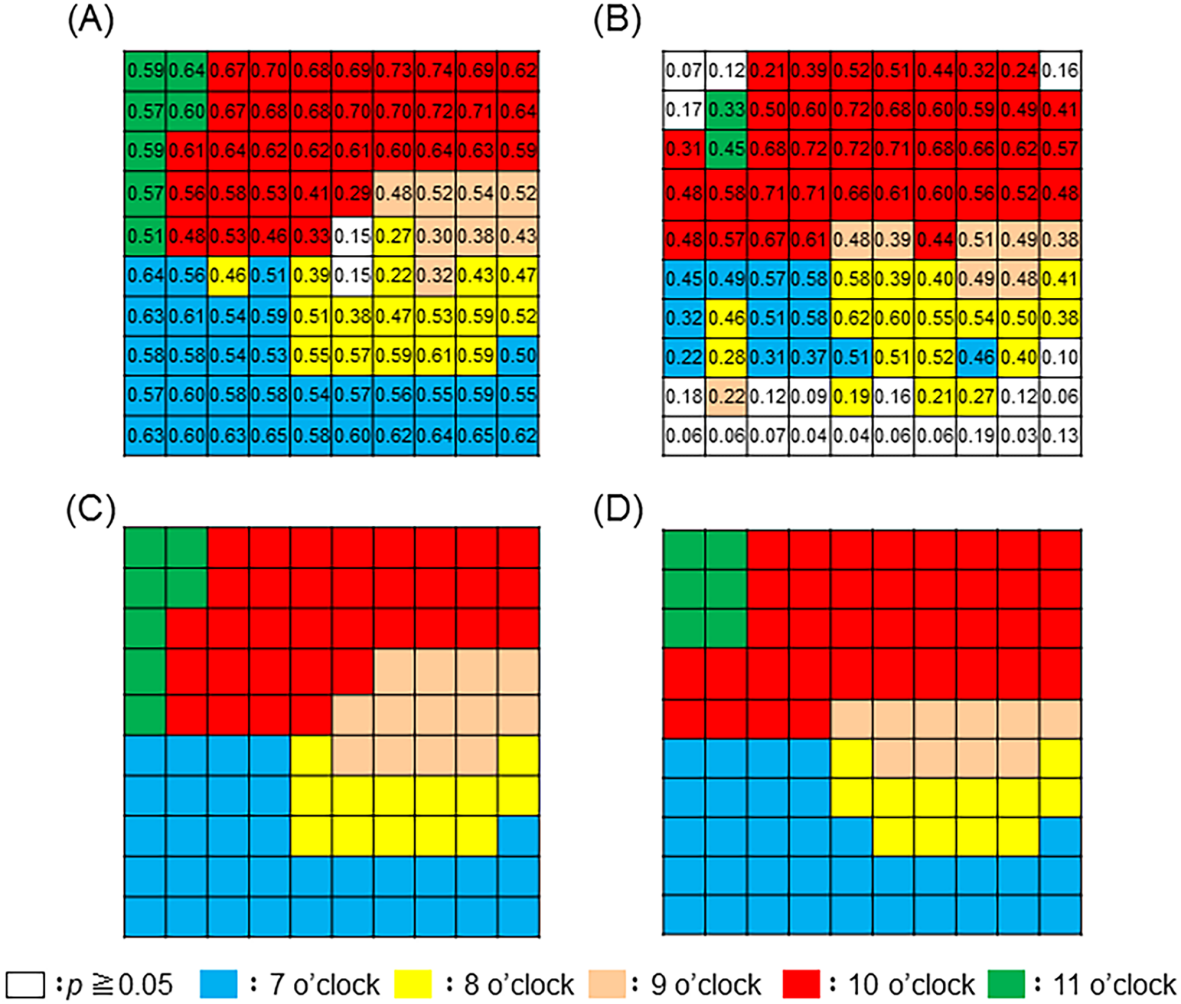
Division of each macular grid point. The distribution of highest correlation between each OCT grid point for mRNFLT (A) and mGCIPLT (B) and each clockwise scan sector (at 7, 8, 9, 10, and 11 o’clock) for cpRNFLT. Also shown is a division of the macular grid points for mRNFLT (C) and mGCIPLT (D).

In the previous study, we examined 101 eyes of 101 patients with mild or moderate NTG and identified axonal tract-dependent macular sectors in the mRNFL and mGCIPL. In the current study, we examined a continuous data set from a newly recruited group of 117 eyes of 117 patients with NTG (55 mild, 32 moderate, and 30 advanced). The characteristics of the NTG participants (n = 117) were as follows: mean age: 60.5 ± 11.4 years; 45 male and 72 female; SE: -3.5 ± 3.0 diopters; IOP: 13.4 ± 3.3 mmHg; HFA-measured mean deviation (MD): -8.3 ± 6.7 dB; pattern standard deviation (PSD): 9.1 ± 4.5 dB; and cpRNFLT: 85.0 ± 14.4 μm. Fig 1A and 1B show which clockwise scan sectors were most highly correlated with mRNFLT and mGCIPLT, respectively, at each grid point. The areas of highest correlation (r ≧ 0.4, p < 0.05) were contiguous and arc-shaped. Next, we corrected these sectors by referring to the anatomical trajectory of the nerve fiber layer (Fig 1C and 1D).

To investigate the potential of the identified axonal tract-dependent sectors to reveal glaucoma progression, we examined a separate group of 122 eyes with NTG. Macular maps from at least 5 OCT examinations (mean: 7.9 ± 1.9 examinations) over a period of at least 2 years (mean: 26.5 ± 2.2 months) were available for all patients. The demographic data for these 122 eyes are shown in Table 1. The average age was 63.4 ± 9.7 and average SE was -2.8 ± 2.6 diopters. Baseline IOP at the time of recruitment into this study was 15.1 ± 2.5 mmHg. HFA-measured MD was -9.0 ± 7.0 dB, and total deviation in the 16 central test points (in a 4 x 4 pattern) was -9.0 ± 7.0 dB. Average cpRNFLT was 82.2 ± 13.7 μm.

**Table 1:** Baseline demographic data at the time of normal-tension glaucoma (NTG) diagnosis.

Demographics	NTG^a^ n = 81 cases, 122 eyes
**Age (years)**	63.4 ± 9.7
**Gender (Female/ Male)**	47 / 34
**Spherical equivalent (D**^b^**)**	-2.8 ± 2.6
**IOP**^c^ **(mm Hg)**	15.1 ± 2.5
**MD**^d^ **(dB)**	-9.0 ± 7.0
**PSD**^e^ **(dB)**	10.2 ± 4.3
**TD c16**^f^ **(dB)**	-9.0 ± 7.0
**MD slope (dB/y)**	-0.7 ± 0.8
**TD c16 slope (dB/y)**	-0.5 ± 0.9
**OCT**^g^ **cpRNFLT**^h^ **(μm)**	82.2 ± 13.7

^a^NTG: normal-tension glaucoma,

^b^D: diopters,

^c^IOP: intraocular pressure,

^d^MD: Humphrey-field analyzer (HFA)-measured mean deviation,

^e^PSD: pattern standard deviation,

^f^TD c16: mean total deviation in the 16 central test points (in a 4 x 4 pattern) of the HFA test,

^g^OCT: optical coherence tomography,

^h^cpRNFLT: circumpapillary retinal nerve fiber layaer thickness.

Data are presented as the mean ± standard deviation.

Baseline mRNFLT and mGCIPLT are listed in Table 2. Baseline mRNFLT and mGCIPLT were both significantly lower in the glaucoma patients than the control subjects. Neither mRNFLT or mGCIPLT showed significant differences in the correlation between values in the overall macula, the superior and inferior hemifields, or the clockwise scan sectors. Average age in the control group (n = 28) was 61.0 ± 5.8 years, matching the study group (Table 1). Average SE was -1.8 ± 2.5 diopters. Baseline IOP was 15.2 ± 2.8 mmHg. HFA-measured MD was 0.4 ± 1.0 dB, PSD was 1.5 ± 0.8 dB, and total deviation in the 16 central test points (in a 4 x 4 pattern) was 0.8 ± 0.9 dB. Average cpRNFLT was 109.8 ± 9.6 μm.

**Table 2:** Baseline macular retinal nerve fiber layer thickness (mRNFLT) and ganglion cell layer plus inner plexiform layer thickness (mGCIPLT) for the sector analysis.

Location	Normal n = 28 cases, 28 eyes	Glaucoma n = 81 cases, 122 eyes	p value
**mRNFLT**^a^ **(μm)**			
**Average**	35.06 ± 3.82	20.66 ± 7.57	<0.001
**Superior**	34.15 ± 3.45	23.08 ± 9.14	<0.001
**Inferior**	35.97 ± 4.99	18.11 ± 9.49	<0.001
**7 o’clock**	37.47 ± 5.92	16.21 ± 10.73	<0.001
**8 o’clock**	37.41 ± 6.15	23.22 ± 10.31	<0.001
**9 o’clock**	29.71 ± 4.61	23.38 ± 7.63	<0.001
**10 o’clock**	37.18 ± 3.78	24.27 ± 10.33	<0.001
**11 o’clock**	21.02 ± 2.40	10.99 ± 8.08	<0.001
**mGCIPLT**^b^ **(μm)**			
**Average**	67.96 ±3.56	55.94 ± 6.14	<0.001
**Superior**	69.24 ± 3.42	57.24 ± 8.09	<0.001
**Inferior**	66.67 ± 4.00	54.94 ± 6.02	<0.001
**7 o’clock**	61.00 ± 4.13	52.18 ± 5.03	<0.001
**8 o’clock**	77.06 ± 4.55	59.56 ± 10.30	<0.001
**9 o’clock**	76.65 ± 6.44	63.37 ± 13.01	<0.001
**10 o’clock**	71.07 ± 3.38	58.19 ± 8.85	<0.001
**11 o’clock**	52.43 ± 3.66	47.97 ± 4.43	<0.001

^a^mRNFLT: macular retinal nerve fiber layer thickness,

^b^mGCIPLT: macular ganglion cell layer plus inner plexiform layer.

The data for average thickness of each layer are presented as the mean ± standard deviation. Differences were considered significant at p < 0.05.

MRNFLT and mGCIPLT slopes are listed in Table 3. The slope of mRNFLT in the 9 o’clock sector (0.001 ± 0.657 μm/year, p < 0.001) was significantly slower than that the overall average mRNFLT slope (-0.22 ± 0.58 μm/year). In each patient, we selected the hemifield with the fastest mRNFLT slope and calculated the average slope in the faster-progressing hemifield (-0.54 ± 0.69 μm/year, p < 0.001). We also selected the temporal clockwise sector with the fastest cpRNFLT slope in each patient and calculated the average slope in the fastest-progressing sector (7 to 11 o’clock, -1.00 ± 0.84, p < 0.001). Both values were significantly faster than the overall average mRNFLT slope. Similarly, the average mGCIPLT slope of the faster-progressing hemifield in each patient was also faster (-0.61 ± 0.53 μm/year, p < 0.001) than the overall mGCIPLT slope, and the average mGCIPLT slope of the fastest-progressing clockwise scan sector was significantly faster (1.16 ± 0.63 μm/year, p < 0.001) than the overall mGCIPLT slope. The overall average mGCIPLT slope was -0.35 ± 0.52 μm/year. The mGCIPLT slope in the superior hemifield (-0.45 ± 0.59 μm/year, p < 0.001) and inferior hemifield (-0.26 ± 0.64 μm/year, p = 0.001) were significantly faster than the overall mGCIPLT slope.

**Table 3:** Comparison of slope of progression in macular OCT parameters.

	mRNFLT^a^		mGCIPLT^b^	
Location	Slope	p value*^c^	Slope	p value**^d^
**Average**	-0.22 ± 0.58	n.c.	-0.35 ± 0.52	n.c. ^e^
**Superior**	-0.18 ± 0.59	0.168	-0.45 ± 0.59	<0.001
**Inferior**	-0.30 ± 0.80	0.094	-0.26 ± 0.64	0.001
**Faster hemifield**	-0.54 ± 0.69	<0.001	-0.61 ± 0.53	<0.001
**7 o’clock**	-0.31 ± 0.95	0.228	-0.13 ± 0.71	<0.001
**8 o’clock**	-0.34 ± 1.00	0.124	-0.63 ± 0.85	<0.001
**9 o’clock**	0.001 ± 0.66	<0.001	-0.48 ± 0.71	0.203
**10 o’clock**	-0.23 ± 0.70	0.833	-0.49 ± 0.66	<0.001
**11 o’clock**	-0.20 ± 0.72	0.605	-0.19 ± 0.78	0.029
**Fastest sector**	-1.00 ± 0.84	<0.001	-1.16 ± 0.63	<0.001
**Fastest overall**	-1.01 ± 0.83	<0.001	-1.16 ± 0.63	<0.001

^a^mRNFLT: macular retinal nerve fiber layer thickness,

^b^mGCIPLT: macular ganglion cell layer plus inner plexiform layer,

^c^*p value: each value was compared with average mRNFLT,

^d^**p value: each value was compared with average mGCIPLT,

^e^n.c.: not compared.

Data are presented as the mean ± standard deviation.

We classified the second group of 122 eyes as VF progressors (PG; 22 eyes) or non-progressors (NPG; 100 eyes) with Progressor software, based on test results in the 16 central test points, i.e., the area corresponding to the OCT macular map. The demographic and average data for the progressors and non-progressors in this study are listed in Table 4. There were no differences between the two groups in sex ratio, age, SE, baseline IOP, cpRNFLT, HFA 24–2 MD, PSD, or total deviation in the 16 central test points (4 x 4). On the other hand, there were significant differences between the two groups in MD slope (p = 0.008) and TD slope in the 16 central test points (p = 0.016).

**Table 4:** Comparison of baseline demographic data at the time of NTG in non-progressors and progressors.

Demographics	Non-progressors n = 70 cases, 100 eyes	Progressors n = 19 cases, 22 eyes	p value
**Age (years)**	63.1 ± 9.9	66.5 ± 7.5	0.089
**Sex (Female/ Male)**	40 / 30	12 / 7	0.794
**Spherical equivalent (D**^a^**)**	-2.9 ± 2.6	-2.7 ± 2.6	0.685
**IOP**^b^ **(mm Hg)**	15.4 ± 2.2	13.8 ± 3.6	0.100
**MD**^c^ **(dB)**	-8.6 ± 6.8	-10.7 ± 7.6	0.185
**PSD**^d^ **(dB)**	10.1 ± 4.4	10.8 ± 4.0	0.433
**TD c16**^e^ **(dB)**	-10.9 ± 7.9	-13.1 ± 8.0	0.323
**MD slope (dB/y)**	-0.7 ± 0.8	-1.2 ± 0.8	0.008
**TD c16 slope (dB/y)**	-0.3 ± 0.8	-1.1 ± 1.0	0.016
**OCT**^f^ **cpRNFLT**^g^ **(μm)**	82.9 ± 13.2	79.1 ± 13.8	0.259

^a^D: diopters,

^b^IOP: intraocular pressure,

^c^MD: Humphrey-field analyzer (HFA)-measured mean deviation,

^d^PSD: pattern standard deviation,

^e^TD c16: mean total deviation in the 16 central test points (in a 4 x 4 pattern) of the HFA test,

^f^OCT: optical coherence tomography,

^g^cpRNFLT: circumpapillary retinal nerve fiber layaer thickness.

Data are presented as the mean ± standard deviation. Differences were considered significant at p < 0.05.

Baseline mRNFLT for the two groups is listed in Table 5. Baseline mRNFLT was significantly different in the two groups in the inferior hemifield (p = 0.032), 7 o’clock sector (p = 0.043), and 8 o’clock sector (p = 0.004). Baseline mGCIPLT was significantly lower in the progressors than the non-progressors in the overall macula (p = 0.039), inferior hemifield (p = 0.015), and 8 o’clock sector (p = 0.017).

**Table 5:** Comparison of baseline OCT parameters at the time of NTG diagnosis in non-progressors and progressors.

Location	Non-progressors (n = 100)	Progressors (n = 22)	p value
**mRNFLT**^a^ **(μm)**			
**Average**	21.23 ± 7.62	17.99 ± 6.89	0.102
**Superior**	23.31 ± 8.90	22.00 ± 10.36	0.583
**Inferior**	18.99 ± 9.69	13.98 ± 7.34	0.032
**7 o’clock**	17.23 ± 11.17	11.10 ± 6.17	0.043
**8 o’clock**	24.40 ± 10.28	16.68 ± 27.95	0.004
**9 o’clock**	23.31 ± 7.59	23.77 ± 8.10	0.755
**10 o’clock**	24.67 ± 10.09	22.37 ± 11.51	0.413
**11 o’clock**	10.98 ± 7.89	11.05 ± 9.11	0.990
**mGCIPLT**^b^ **(μm)**			
**Average**	56.45 ± 6.30	53.01 ± 4.11	0.039
**Superior**	57.71 ± 8.24	54.48 ± 6.76	0.121
**Inferior**	55.52 ± 6.17	51.54 ± 3.52	0.015
**7 o’clock**	52.32 ± 5.19	51.47 ± 4.14	0.523
**8 o’clock**	60.57 ± 10.25	54.24 ± 9.12	0.017
**9 o’clock**	63.82 ± 12.71	61.03 ± 14.66	0.312
**10 o’clock**	58.53 ± 8.95	56.49 ± 8.36	0.387
**11 o’clock**	48.02 ± 4.48	47.69 ± 4.27	0.993

^a^mRNFLT: macular retinal nerve fiber layer thickness,

^b^mGCIPLT: macular ganglion cell layer plus inner plexiform layer.

Differences were considered significant at p < 0.05.

A trend analysis showed that the mRNFLT slope in the faster-progressing hemifield (p = 0.045), the 8 o’clock sector, (p = 0.046), and the fastest-progressing clockwise sector (among the 7 to 11 o’clock sectors) (p = 0.022) was significantly faster in the progressors than the non-progressors (shown in Table 6). However, mGCIPLT slope did not significantly differ in the progressors and non-progressors.

**Table 6:** Comparison of trend analysis results in non-progressors and progressors.

Location	Non-progressors(n = 100)	Progressors(n = 22)	p value
**mRNFLT**^a^ **slope (μm/Y)**			
**Average**	-0.17 ± 0.54	-0.47 ± 0.70	0.191
**Superior**	-0.16 ± 0.55	-0.28 ± 0.77	0.522
**Inferior**	-0.22 ± 0.73	-0.66 ± 0.98	0.120
**Faster hemifield**	-0.47 ± 0.61	-0.90 ± 0.89	0.045
**7 o’clock**	-0.24 ± 0.88	-0.60 ± 1.19	0.241
**8 o’clock**	-0.24 ± 0.95	-0.79 ± 1.09	0.046
**9 o’clock**	0.04 ± 0.61	-0.19 ± 0.81	0.101
**10 o’clock**	-0.20 ± 0.65	-0.37 ± 0.89	0.503
**11 o’clock**	-0.17 ± 0.73	-0.31 ± 0.70	0.425
**Fastest sector**	-0.89 ± 0.76	-1.47 ± 1.03	0.022
**Fastest sector for mRNFLT**	-0.91 ± 0.75	-1.48 ± 1.03	0.025
**mGCIPLT**^b^ **slope (μm/Y)**			
**Average**	-0.36 ± 0.52	-0.31 ± 0.51	0.919
**Superior**	-0.45 ± 0.62	-0.45 ± 0.44	0.951
**Inferior**	-0.28 ± 0.62	-0.16 ± 0.73	0.619
**Faster hemifield**	-0.61 ± 0.54	-0.59 ± 0.48	0.960
**7 o’clock**	-0.15 ± 0.69	-0.02 ± 0.82	0.717
**8 o’clock**	-0.68 ± 0.76	-0.40 ± 1.17	0.287
**9 o’clock**	-0.46 ± 0.71	-0.54 ± 0.78	0.627
**10 o’clock**	-0.50 ± 0.69	-0.49 ± 0.54	0.978
**11 o’clock**	-0.18 ± 0.82	-0.25 ± 0.58	0.559
**Fastest sector**	-1.15 ± 0.62	-1.21 ± 0.65	0.924
**Fastest sector for mGCIPLT**	-1.15 ± 0.63	-1.22 ± 0.65	0.852

^a^mRNFLT: macular retinal nerve fiber layer thickness,

^b^mGCIPLT: macular ganglion cell layer plus inner plexiform layer.

Differences were considered significant at p < 0.05.

**Fig 2:**
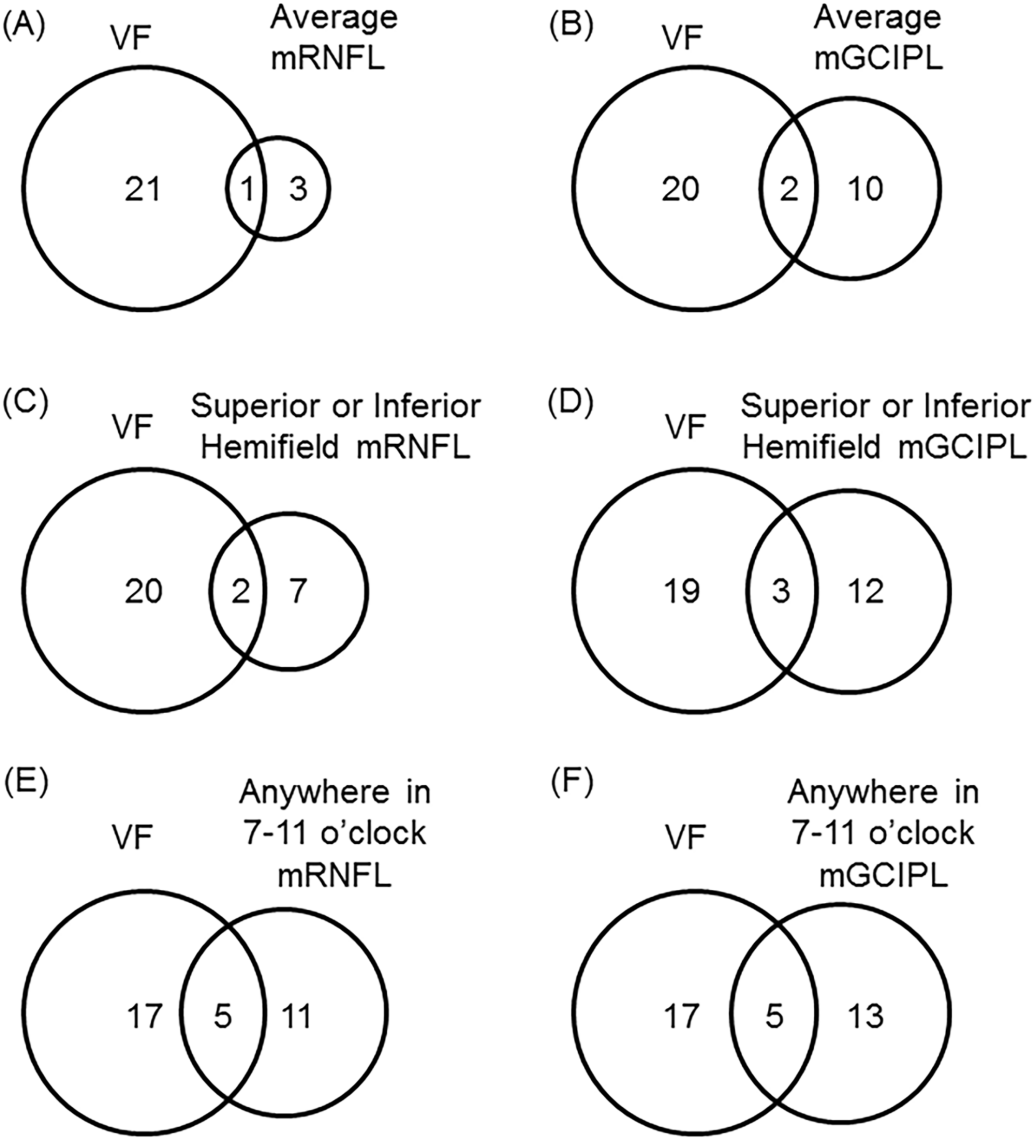
Glaucoma progression. Venn diagrams showing glaucoma progression as evaluated with a visual field (VF) analysis and with OCT. (A) Average mRNFLT progression, (B) average mGCIPLT progression, (C) mRNFLT progression in any hemifield, (D) mGCIPLT progression in any hemifield, (E) mRNFLT progression in any clockwise sector, (F) mGCIPLT progression in any clockwise sector.

Table 7 shows the results of the event analysis. In the overall group of patients (n = 122), mRNFLT progression occurred in 4 eyes in the overall average macular map, in 1 eye in the superior hemifield, and in 8 eyes in the inferior hemifield. Among the 7–11 o’clock sectors, mRNFLT progression occurred in 16 cases. In the same group, mGCIPLT progression occurred in 12 eyes in the overall average macular map, in 15 eyes in the superior hemifield, and in 2 eyes in the inferior hemifield. Among the 7–11 o’clock sectors, mGCIPLT progression did not occur in any case in the 7 o’clock sector; it occurred predominantly in the 10 o’clock sector, in 15 eyes. In total, mGCIPLT progression occurred in 18 eyes in the temporal clockwise sectors. A comparison of the results of the event analysis in the overall average macular map, hemifields, and clockwise sectors showed that the clockwise sectors provided results that were most consistent with those of the VF progression analysis performed with Progressor software (shown in Fig 2).

**Table 7:** Comparison of event analysis results in the overall group, the non-progressors, and the progressors.

Location	Overall (n = 122)		Non-progressors (n = 100)		Progressors (n = 22)	
mRNFLT^a^	(eyes)	(%)	(eyes)	(%)	(eyes)	(%)
**Average**	4	3.3	3	3.0	1	4.5
**Superior**	1	0.8	1	1.0	0	0
**Inferior**	8	6.6	6	6.0	2	9.1
**Progression in any hemifield**	9	7.4	7	7.0	2	9.1
**7 o’clock**	7	5.7	5	5.0	2	9.1
**8 o’clock**	3	2.5	2	2.0	1	4.5
**9 o’clock**	0	0	0	0	0	0
**10 o’clock**	6	4.9	4	4.0	2	9.1
**11 o’clock**	1	0.8	1	1.0	0	0
**Progression in any sector**	16	13.1	11	11.0	5	22.7
**mRNFLT progression in any sector**	17	13.9	12	12.0	5	22.7
**mGCIPLT**^b^						
**Average**	12	9.8	10	10.0	2	9.1
**Superior**	15	12.3	12	12.0	3	13.6
**Inferior**	2	1.6	2	2.0	0	0
**Progression in any hemifield**	15	12.3	12	12.0	3	13.6
**7 o’clock**	0	0	0	0	0	0
**8 o’clock**	5	4.1	3	3.0	2	9.1
**9 o’clock**	3	2.5	2	2.0	1	4.5
**10 o’clock**	15	12.3	12	12.0	3	13.6
**11 o’clock**	1	0.8	1	1.0	0	0
**Progression in any sector**	18	14.8	13	13.0	5	22.7
**mGCIPLT progression in any sector**	20	16.4	15	15.0	5	22.7

^a^mRNFLT: macular retinal nerve fiber layer thickness,

^b^mGCIPLT: macular ganglion cell layer plus inner plexiform layer.

## Discussion

In this study, we set out to determine whether specific, axonal tract-dependent sectors of the OCT macular map could be used to effectively measure the progression of structural changes in glaucoma, operating on the hypothesis that glaucomatous changes predominantly occur in areas of the retina that depend on the axonal tract. We identified axonal tract-dependent sectors in the macula and investigated the role of these sectors in glaucomatous damage with trend and event analyses of longitudinal changes over 2 years. We found that mRNFLT and mGCIPLT progression occurred with a more rapid slope and more often in patients with VF progression. These findings suggest that the new macular map sectors described in this study should help improve the assessment of progression in patients with NTG.

The gold standard to detect glaucoma progression remains the assessment of functional changes, but structural changes in glaucoma are also progressive. Previously, red-free fundus photography has been shown to clearly reveal the progression of glaucoma. Long-term clinical observation of patients with preperimetric NTG has shown that 70% undergo widening of retinal nerve fiber layer defects (RNFLDs) and 33% undergo deepening of the RNFLDs, suggesting that the angle of the RNFLDs may be an effective marker for diagnosis and evaluation of VF progression. [32,33] CpRNFLT is also a sensitive marker of glaucoma progression. A joint longitudinal survival model showed that a 1 μm/year faster rate of RNFLT loss corresponded to a 2.05-times higher risk of developing visual field defects. [22] In this study, we have extended these findings to show that macular thickness in specific sectors of the macular map can also be used to evaluate glaucoma progression with high sensitivity. These findings suggest that the measurement of macular structure may also be a valuable way to evaluate glaucoma.

The sectoral nature of changes in HFA-measured MD is of fundamental importance for understanding glaucoma, because the pattern of changes reflects the axonal damage that occurs in the lamina cribrosa during glaucoma, and corresponds with the characteristic arcuate shape of the axonal tract-dependent areas of the RNFL. Thus, glaucomatous visual field loss is co-localized with the preceding axonal injury. This has led to various strategies to improve the diagnosis of glaucoma by studying different sectors of the retina. [34,35] Garway-Heath et al. established the anatomical relationship between regional visual field sensitivity, measured with the HFA 24–2 program, and rim thinning in the optic nerve head. [36] Another group used a mathematical approach to identify important sectors in HFA 30–2 program results. [37,38] Recently, progress in OCT technology has enhanced the potential to study structural changes in the macula and optic nerve head in glaucoma. Investigation of the relationship between structure and function in glaucoma has shown that there is a regional relationship between OCT-measured cpRNFLT and visual field sensitivity. [39] However, the sectoral nature of changes in the OCT macular map has not yet been investigated. Generally, glaucoma tends to affect the inner retinal layers, particularly the RNFL, GCIPL, and ganglion cell complex. Thus, macular structural assessment promises to improve the detection of glaucoma through examination of the different layers of the retina.

Thus, the aim of the current study was to identify sectors of the macula in OCT scans that could effectively reveal the progression of glaucoma. Visual field sectors of the 10–2 program that can reveal progression have already been described, and previous studies have also reported methods to identify important sectors in the OCT macular map by comparison with regional HFA 10-2-measured visual field sensitivity. However, structural changes precede visual field loss in glaucoma, and fundus and test points are adjusted depending on the position of the ONH in relation to the fovea, and depending on disc area, axial length, spherical equivalent, disc shape, disc orientation, and disc tilt. [40] Here, we minimized the possibility of bias caused by these factors by comparing the anatomical relationship between the macular and disc areas, i.e., the OCT-measured macular map and cpRNFLT, respectively.

The sectors of the macula that we identified as important may help to diagnose glaucoma and to evaluate glaucoma progression, and may be most useful as part of a cluster analysis. Recently, Kanamori et al. [41] and Raza et al. showed that identifying contiguous clusters of abnormal points can improve the detection of glaucoma. [42] Thus, accurate information on the relationship between specific areas of the macular map and the anatomical trajectory of the nerve fibers would be a valuable addition to future investigations of the potential of the macular map to help diagnose glaucoma and evaluate the structural progression of the disease, and should improve the sensitivity and specificity of tests for glaucoma.

An interesting finding of this study was that the area of the macula with the highest correlation to peripapillary thickness differed for mRNFLT and mGCIPLT (Fig 1). One reason for this difference may lie in the displacement of the RGCs within 7.2 degrees of the macula, which causes the absolute thickness of the mRNFL and mGCIPL to differ. [43] Another reason may be differing patterns of damage in the mRNFL and mGCIPL. Therefore, thinning of the mGCIPL may represent *in situ* damage to the RGCs, while on the other hand, thinning of the mRNFL may represent the sum total of damage to the projections of the retinal nerve fibers. The combination of a torus-shaped pattern of mGCIPL damage and the high frequency of damage to the inferior temporal rim of the optic disc may underlie changes in infero-temporal mGCIPL. [34,35] While the mRNFL is thicker towards the optic disc, the mGCIPL shows no large variations within the macular map. These differences in the topographical characteristics of the mRNFL and mGCIPL in the OCT macular map may thus influence the sectoral nature of damage in these two layers.

The current study found that overall, the slope of progression in the fastest-progressing sectors of the mRNFL and mGCIPL was similar (Table 3). However, dividing the patients according to whether they underwent visual field progression revealed significant differences in the slope of progression in the mRNFL and mGCIPL (Table 5). Specifically, mRNFLT had a significantly different slope in the progressors and non-progressors, but mGCIPLT did not. Generally, the pathogenesis of glaucoma begins with axonal damage in the lamina cribrosa, which proceeds to functional loss. However, it currently remains unclear whether axonal damage or cell body death of the RGCs is earlier in human glaucoma. In mice that undergo experimental optic nerve axotomy, dendrite degeneration proceeds to cell body loss; furthermore, the somal and axonal degeneration pathways are different in DBA/2J mice. [44] Somal degeneration depends on the mitochondrial pathway, but axonal loss does not, and is prevented by the Wld(s) allele. [45] Still, it is possible that different types of glaucoma pathogenesis, such as high IOP, oxidative stress, ischemia, and mitochondrial dysfunction, cause different types of damage. Our findings may thus suggest that it is reasonable to evaluate mRNFLT and mGCIPLT separately. We found no significant differences in mGCIPLT in the progressive and non-progressive groups in this study, but this finding may have been due to the differing stages of glaucoma among our patients. Further study is needed to determine the most efficient way to evaluate glaucoma progression at different stages of the disease.

The trend and event analyses of the hemifields shown in Tables 3 and 7 indicate that even though the speed of progression was similar in the faster-progressing hemifield, progression occurred more often in the inferior mRNFL and superior mGCIPL. This result may indicate that regional susceptibility varies in NTG patients. Recently, the diagnostic value of hemifield differences in mGCIPLT, i.e., above and below the horizontal raphe, has been shown to be excellent even in PPG. [46,47] These studies showed that in PPG, inferior mGCIPLT begins to decrease, and when glaucoma reaches the perimetric stage, inferior mGCIPLT has already reached a floor level. Finally, superior mGCIPLT begins to decrease only in the later stages of glaucoma. However, this process does not correlate to VF progression in the macular area. This may be because different stages of glaucoma have different characteristics in the range of changes in mGCIPLT. This suggests that the current study, which examined changes in OCT measurements over a period of 2 years, might have had different findings if the study design had a period of more than 5 years and had included patients with PPG. Such a design might have revealed stage-dependent changes in mRNFLT and mGCIPLT. Thus, further study of this issue is required.

This study had several limitations. The first was the difficulty of evaluating the speed of glaucomatous changes when they occurred outside macular lesions. Lisboa et al. showed that for the OCT-based detection and diagnosis of PPG, cpRNFLT is more accurate than parameters of macular layer thickness. [48] However, a previous hospital-based study published by us showed that 75% of NTG patients had decreased mRNFLT in macular lesions. [49] Furthermore, in Asia, the major type of open-angle glaucoma is NTG, which has more macular lesions than POAG. [10,11] Kim et al. [50] showed that inferior mGCIPLT loss might occur earlier than cpRNFLT loss in a macular vulnerability zone. [34,35] This implies that macular thickness assessments may be more useful in Asian patients. A second limitation of this study was a relatively small study population in the progression study, which may have affected the statistical power of our analyses. Third, we could not avoid certain factors, such as myopia and age, that might have biased our OCT data on retinal layer thickness. [51] Nevertheless, our evaluation of sectoral differences in the slope of progression was based on inter-individual assessments, and the influence of these factors should have thus been minimal.

In conclusion, in this study, we identified macular sectors that depend on the axonal tract in the retina, and determined the statistical association between macular layer thickness in these sectors and cpRNFLT. This showed that when the sector with the fastest progression in mRNFLT and mGCIPLT loss was selected, the slope of progression was significantly faster than the slope in the overall OCT macular map. These findings show that analyzing axonal tract-dependent sectors in the macular map may help improve the accuracy of clinical assessments of NTG progression.

## Supporting information

S1 File OCT measurement for axonal tract-dependent macular sectors. F1 File contains OCT measurement of mRNFLT and mGCIPLT.
(XLSX)
